# The Effects of Childhood Circumstances on Health in Middle and Later Life: Evidence From China

**DOI:** 10.3389/fpubh.2021.642520

**Published:** 2021-02-03

**Authors:** Tiantian Tao, Rong Shao, Yuanjia Hu

**Affiliations:** ^1^School of International Pharmaceutical Business, China Pharmaceutical University, Nanjing, China; ^2^State Laboratory of Quality Research in Chinese Medicine, Institute of Chinese Medical Sciences, University of Macau, Macau, China

**Keywords:** childhood circumstances, healthy aging, life-course model, panel data, health disparity, economic burden, middle-aged and elderly population

## Abstract

**Background:** This study examined the relationship between childhood circumstances and health in middle and later life. We quantified how childhood circumstances contribute to health in later life, both directly and indirectly, through their effects on potential mediators.

**Methods:** This study used three waves of data from the national longitudinal survey of the China Health and Retirement Longitudinal Study (CHARLS). The final model in this study included 7,476 eligible respondents aged 45 years and above. We constructed a simple health status measure based on the first principal component of CHARLS survey responses with 25 health-related information. It is a multi-dimensional measurement that comprehensively reflects the individual's healthy aging. We formulated childhood circumstances factors into five domains: childhood health and nutrition, childhood socioeconomic status, access to health care, parental genetics, and adverse childhood experiences. Ordered logit regression was conducted to analyze the relationship between health in middle and later life and childhood circumstances, with other explanatory variables controlled.

**Results:** Controlling for educational attainment, personal income, and health status in the last wave, adults who experience good childhood health (poor as the base, coefficient 0.448, *p* < 0.01), and better family financial status (worse as the base, coefficient 0.173, *p* < 0.01) have significantly better health during their middle and later life, in comparison, being inconvenient to visit a doctor (coefficient −0.178, *p* < 0.01), and having two or three adverse childhood experiences (0 as the base, coefficient −0.148, *p* < 0.01) are significantly associated with poorer health. Childhood circumstances appear to act both through a lasting effect of initial health and financial status in childhood and through their impact on achievements in adulthood.

**Conclusion:** Our findings suggest that investments in health during childhood not only contribute to health in later life but also dynamically improve an individual's educational attainment and personal income, as well as other life prospects. All these returns may extend far beyond childhood and continue throughout the lifespan.

## Introduction

The phenomenon of population aging has become a global trend (1–3). In 2017, the global population over 60 years old reached 962 million, and this number is expected to increase to 2.1 billion by 2050. China is facing a more difficult aging situation (4). In 2017, one in four of the world's total aging population lived in China, and the proportion of the elderly population in China was 16%. The proportion has changed recently and is expected to further increase to 35% by 2050 (3). A significant contributor to the aging population is that expected longevity has increased dramatically. Currently, life expectancy at birth is at least 77.0 years in China, and it increased by 75% between 1960 and 2015 (5). However, a longer lifespan does not necessarily mean that one's quality of life also improves. The physical, psychological, and cognitive health problems associated with aging are still a concern (6). In 2018, China's quality-adjusted life expectancy per capita was only 68.7 years. The elderly survived an average of more than 8 years with diseases, and the number of functionally limited and partially limited elderly exceeded 40 million. Among the elderly, the proportion of patients suffering from more than one chronic disease was as high as 75%, which accounted for 190 million people (7). For this reason, promoting and achieving healthy aging has become a critical public health issue in China. In particular, most of the health problems mentioned above do not come across suddenly but act as a cumulative process. That also prompts us to pay attention to the link between experiences from earlier life stages and healthy aging.

As an essential part of human capital, health is regarded as a durable product throughout the life cycle since Grossman published his seminal work on the demand for health (8). At present, many research groups continue their work on the long-term impact of childhood circumstances on future health, as well as social life (9, 10). The available evidence indicates that returns on health investment during childhood are higher than in other life stages (11). Recent studies have demonstrated that a wide range of childhood circumstances may lead to health disparity in adulthood (12–14), such as health and nutrition (15), parents' education levels and family socioeconomic status (16, 17), early life development and genetic inheritance (18, 19), adverse childhood experiences (12, 20), and access to medical services in the community (21). Some of these studies have found that adverse shocks during childhood can adversely affect adult health, while other studies have demonstrated the positive effects of childhood interventions on future health (21, 22). However, prior work is generally limited to a subset of childhood circumstances. It is necessary to discuss all these domains of childhood circumstances in the same model, as well as consider that domains may be inter-related.

Previous studies have suggested several pathway models to explain the correlation between childhood circumstances and health. Some life course models emphasize that childhood circumstances have either direct or indirect effects on adult health (17, 23). The direct effects work through the adverse experience itself. The indirect effects limit educational achievements and other life-related better changes, making it challenging to maintain a healthier life in adulthood. Evidence from developed countries has revealed a common phenomenon: children who grow up in a more impoverished environment have worse health outcomes as they age than those who do not, and their educational attainments and labor income tend to be lower (16, 24–26). Furthermore, some studies have explored the indirect effects in greater depth. These studies focused on the role of socioeconomic status in determining adult health and concluded that the association between social achievements and health in later life could not be simply attributed to childhood experiences. Circumstances in early life influence adult circumstances, which, in turn, affect the development of diseases (27–29). The fetal origins hypothesis suggests that malnutrition and health shocks in early life can cause hidden developmental problems in the immune system and vital organs (30, 31). Thus, they directly affect one's health status in all stages of life. This hypothesis also suggests another possible pathway. Children born into poor families are more likely to be exposed to a poor fetal environment, so their future health status is poorer than those in wealthy families. Their future socioeconomic status is also likely to be lower. As a result, the above-mentioned correlation may also appear between socioeconomic status and health in adulthood. From the life-course perspective, it is of great significance to explore how childhood circumstances affect health in middle and later life, especially the dynamic relationship between health and socioeconomic status.

Although some attempts have been made to address the issue of how childhood circumstances influence health status when individuals get old, some of the aspects require further research. First, previous studies of childhood circumstances typically have not included a large number of health outcomes. Most of them examined a single disease or one particular domain of health status, such as acute myocardial infarction (32), or various psychological measures (14). They are useful in estimating prevalence, but these results limit the development of a more comprehensive definition of healthy aging. People are gradually giving new meanings to health, and the multi-dimensional concept of health has been widely recognized by academia (33). Health is not merely the absence of disease or infirmity, but a state of complete physical, mental, and social well-being (34). Specifically, Healthy aging does not only mean an increase in the average life expectancy, but should also focus on all aspects of the quality of life, which is the perfect status in terms of morality, freedom, and happiness. Second, the causal pathway between health and socioeconomic status has not been demonstrated (9, 35), which makes it more complicated to study the pathway from childhood circumstances to health in middle and later life. Slightly fewer attempts at examining these possible pathways in the same research framework have been reported in the literature. Third, most of the existing studies were conducted in developed countries, and the conclusions are not necessarily applicable to populations in developing countries. Compared with these studies, our study has the following two main contributions. First, we have defined a health index that contains various aspects of healthy living information, which measures the degree of an individual's healthy aging. Second, experiences in middle and high-income countries, such as China, may bring more inspiration to developing countries. The generation of Chinese represented by this research sample has undergone a complicated social change process: the period from the early days of the founding of the People's Republic of China with low-income levels to modern times' rapid development (15, 36). This process can reflect the overall operating law of changes in socioeconomic status and people's health in developing countries. Our study aims at an in-depth understanding of the above issue. We quantified the long-term effects of childhood circumstances on health status in middle and later life, using the ordered logit model based on data from three waves of China's Health and Retirement Longitudinal Study (CHARLS) (37). Due to the difficulty in making causal inferences, we began by presenting reduced-form estimates of the association between childhood circumstances and health in middle and later life. Then, we considered several pathway models to determine whether childhood circumstances directly affect future health and how these circumstances contribute to the dynamic relationship between health and socioeconomic status in middle and later life.

## Methods

### Data and the Sample

Data were collected in 2013 and 2015 for CHARLS, as well as in 2014 for a life history survey conducted by the National School of Development at Peking University. CHARLS is a longitudinal study of individuals over age 45. The first wave was conducted in 2011, and follow-up surveys have been conducted every 2 years after that. In 2014, CHARLS included a life-history survey that asked questions about the life experiences of respondents since birth, including their migration and residential history, economic and social conditions in childhood, and so on. For our study, variables associated with childhood circumstances were extracted from the 2014 survey results. Health status and socioeconomic data were obtained from the 2013 and 2015 waves. CHARLS covers all county-level units in mainland China (excluding Tibet, Hainan, and Ningxia). For example, the 2015 sample includes 150 county-level and 450 village-level units, with a total of 11,797 households (20,284 individuals). The survey provides rich data regarding the physical and psychological health, demographics, and socioeconomic status of middle-aged and older adults. More detailed information about CHARLS can be obtained from Peking University's website (38, 39).

### Sample Selection Criteria

Our study included individuals who participated in the 2014 survey and responded in either 2013 or 2015 surveys, and we also excluded individuals aged under 45. First, we used the 2014 sample to match the 2015 or 2013 data and obtained two sub-samples for the correlational study. We also deleted observations for individuals under age 45 or with missing values. Then, we merged the data from all three waves and used the 2014 records as the primary sample. Since CHARLS collects unbalanced panel data, there are cases of sample loss and new sample enrollment in each fellow-up wave, so the final three-wave, balanced sample size in our final analysis is 7,636.

### Measurement

#### Health Status Index

We constructed a health index to measure each respondent's health status in the different waves. CHARLS asks respondents a large number of health-related questions. We used 25 of these questions (see Table 1), including the following: (i) an indicator of whether the respondent has a poor health status, as measured by self-reported health; (ii) indicators of whether the respondent suffers from major diseases, including 14 medical conditions (hypertension, dyslipidemia, diabetes, cancer, chronic lung diseases, liver disease, heart problems, stroke, kidney disease, digestive disease, psychiatric problems, memory-related disease, arthritis, and asthma); (iii) indicators of whether the respondent has any functional limitations in daily life, measured over five instrumental activities of daily living (i.e., performing household chores, preparing meals, shopping, managing money, and taking medication); (iv) indicators of cognition and depressive symptoms, measured by the respondent's self-reported memory level and the Center for Epidemiologic Studies Depression Scale-10 [CESD-10; individuals with a total score of 10 or higher were considered to have a high risk of depression (23, 40)]; (v) an indicator of physical pain measured by the presence of self-reported pain in various parts of the body; (vi) an indicator of participation in social activities, measured by whether the respondent had participated in 11 daily social activities in the past month.

**Table 1:** Health index weights.

**Variable**	**Loading**	**KMO**
Difficulty performing household chores	0.3462	0.7839
Difficulty preparing meals	0.3350	0.7685
Difficulty shopping for groceries	0.3216	0.8474
Self-reported health fair or poor	0.3160	0.8783
Difficulty managing money	0.2933	0.8429
High risk of depression	0.2763	0.8614
Troubled with physical pains	0.2681	0.8363
Difficulty taking medications	0.2256	0.8834
Self-reported memory fair or poor	0.2181	0.8569
Ever experience arthritis	0.1957	0.8399
Ever experience heart problems	0.1711	0.8212
Ever experience chronic lung diseases	0.1492	0.6814
Ever experience stomach disease	0.1491	0.8183
Ever experience hypertension	0.1306	0.7471
Ever experience stroke	0.1259	0.8582
Ever experience kidney disease	0.1219	0.8474
Ever experience memory-related disease	0.1179	0.8747
Ever experience asthma	0.1149	0.6285
Ever experience mental disease	0.1068	0.8834
Ever experience dyslipidemia	0.1024	0.7029
Ever experience diabetes	0.0955	0.7288
Ever participate in any social activity during last month	0.0818	0.8256
Ever experience liver disease	0.0785	0.7897
Ever experience cancer	0.0329	0.7796
Overall	—	0.8114

*The variables in the table are ordered by the principal component loadings*.

#### Childhood Circumstances Factors

The selection of childhood circumstances variables was guided by previous studies, which led us to formulate them into five domains.

Childhood health and nutrition were measured with two variables: (i) self-reported health status before age 15 (categorized as poor, fair, or good); and (ii) ever experienced hunger from birth to age 5 (15, 41).

Childhood socioeconomic status was measured with three variables: (i) family financial status before age 17 (categorized as worse, fair, or better); and (ii) parents' educational attainment (measured by years of education) (27, 42).

Access to health care was measured with two variables: (i) received any vaccinations before age 15; and (ii) experienced inconvenience in visiting a doctor from birth to age 15 (43).

Parental genetics were measured with four variables: (i) parents' longevity (for parents who have passed away, measured as their age at death, and for parents who are alive, measured as their current age); and (ii) whether the respondents' parents are still living (indicators of mother or father still living) (13, 41).

Adverse childhood experiences (ACE) were collected from the answer to 11 questions, including the following: (i) abuse (indicators of often or sometimes being hit by one's father, mother, or other children); (ii) parental depression (indicators of whether the respondent's mother or father displayed ongoing depressive symptoms during most or all of his/her childhood); (iii) parental addiction problems (indicators of whether the respondent's mother or father has had problems with alcoholism, drug addiction, or gambling); (iv) parental marital conflict (an indicator of a fair or poor relationship between the respondent's parents, and whether the parents often or sometimes quarreled); (v) indicators of a fair or poor relationship between child and father or between child and mother. The available evidence indicates that experiencing one ACE category is highly correlated with experiencing one or more of the others (12, 44). In line with these studies, an ACE count was also used to classify respondents into four categories: 0 ACEs, 1 ACE, 2-3 ACEs, and 4+ ACEs.

#### Other Explanatory Variables

The respondents' current socioeconomic status was measured with two variables: (i) educational attainment, measured by years of education, and (ii) personal annual income, measured by the sum of personal labor-related annual income and household asset annual income per capita. Then, the respondent's annual income was categorized as below or above the median (23). Age and gender were included as explanatory variables in all models.

### Analytical Strategy

Our health status index measures the weighted average score respondents achieve across all six domains, and higher values indicate a healthier status. After standardizing the data for those 25 health measures mentioned above, we used principal component analysis to reduce dimensionality. We took the first principal component as the health status index, following previous studies (9, 45). The first principal component is the weighted average of these health indicators, where the weights were chosen to maximize the proportioare as follows. First, then of the variance of health indicators that can be explained by this weighted average. So that index is a standardized variable with a mean of 0 and a variance of 1. We also tested the robustness of the measurement between different subgroups, such as male and female groups, as well as data collected during different waves. However, the results were similar, so we combined all waves and both male and female groups in the same analysis. Finally, we converted the health status index into five quantiles, which can sequentially distinguish populations at different health levels.

Our primary analysis was aimed at estimating the independent effect of childhood circumstances on health status in middle and later life while accounting for the role of the individual's current socioeconomic status and health status in the previous period. To achieve this goal, our analysis was divided into three stages. First, we estimated the reduced form equation, which examined the association between childhood circumstances and health status in middle and later life after controlling for demographic variables. Second, we added contemporary socioeconomic variables to the reduced form equation estimated in the first step, which explored the indirect pathways. In this model, if the coefficients of childhood circumstances changed significantly, then it indicated the existence of an indirect effect; however, if the coefficients did not change much, it demonstrated a direct effect. Third, we added the individuals' previous health status to the model in the second step, which verified the stability of the lasting effect of childhood circumstances and explored the possibility of other potential mediators (46, 47).

## Results

### Sample Description

The principal component loadings and KMO (Kaiser-Meyer-Olkin) measures of variables are shown in Table 1. The health status index assigns higher weights to IADLs, self-reported health, cognition and depressive symptom, and body pains. Much fewer weights are given to specific health problem experiences and social activities participation. The overall KMO value is 0.8114, which means that the commonality among variables is strong, and the analysis can play a better data reduction effect. The variables used in our analysis are summarized in Table 2. Each of the samples in Table 2 corresponds to different panel data combinations: 2013–2014, 2014–2015, and 2013–2015. The first two combinations were used to test the association between childhood circumstances and health status in middle and later life, while the last one was used to explore potential pathway mechanisms. The data in each sample were structured consistently based on the mean and variance of key variables. We can use the third sample to describe the population as follows. In the sample, there are slightly more women (0.4% more) than men, with an average age of 59.92 years. The respondents had an average education level of 6.15 years (equivalent to primary school graduation), their fathers had an average of 2.43 years of education, while their mothers had an average of 0.74 years. In 2015, the annual income per capita was 10,276.53 yuan. Most individuals reported that their family's financial situation prior to age 17 was worse, while most individuals reported that their health status before age 15 was good. In the sample, 35.2% of the respondents experienced food scarcity at some time between birth and age 5, and 12.9% did not receive any vaccinations prior to age 15. However, 34.68% reportedly had not been exposed to the 11 serious adverse events defined above, and the mothers' average life expectancy and total alive number were higher than those of the fathers'.

**Table 2:** Descriptive statistics.

**Variable**	**2014–2015 sample**		**2013–2014 sample**		**2013–2015 sample**	
	**Mean (S.D.)**	**Observations**	**Mean (S.D.)**	**Observations**	**Mean (S.D.)**	**Observations**
**Individual characteristics**						
Female	0.503 (0.500)	10,934	0.514 (0.500)	9,489	0.504 (0.500)	7,476
Age in 2015	60.088 (8.806)	10,934			59.920 (8.413)	7,476
Age in 2013			58.891 (8.849)	9,489		
**Childhood health and nutrition**						
Self-reported health status, before age 15 (1-poor,2-fair,3-good)	2.250 (0.655)	10,934	2.255 (0.652)	9,489	2.266 (0.651)	7,476
Ever experienced hunger, from birth to age 5	0.354 (0.478)	10,934	0.347 (0.476)	9,489	0.352 (0.478)	7,476
**Childhood socioeconomic status**						
Family financial status, before age 17 (1-worse,2-fair,3-better)	1.727 (0.627)	10,934	1.727 (0.623)	9,489	1.728 (0.619)	7,476
Mother's educational attainment	0.776 (2.164)	10,934	0.693 (2.043)	9,489	0.738 (2.090)	7,476
Father's educational attainment	2.475 (3.410)	10,934	2.340 (3.329)	9,489	2.436 (3.357)	7,476
**Access to health care**						
Received any vaccinations, before age 15	0.871 (0.335)	10,934	0.859 (0.348)	9,489	0.871 (0.335)	7,476
Experienced inconvenience in visiting a doctor, from birth to age 15	0.089 (0.284)	10,934	0.091 (0.288)	9,489	0.087 (0.282)	7,476
**Parental genetics**						
Mother's longevity	75.092 (13.328)	10,934	75.348 (13.408)	9,489	75.389 (13.318)	7,476
Father's longevity	73.070 (12.980)	10,934	73.070 (13.140)	9,489	73.212 (12.992)	7,476
Mother still living	0.228 (0.419)	10,934	0.288 (0.453)	9,489	0.257 (0.437)	7,476
Father still living	0.126 (0.332)	10,934	0.165 (0.371)	9,489	0.146 (0.353)	7,476
**Adverse childhood experiences (%)**						
0 ACEs	34.06	10,934	34.14	9,489	34.68	7,476
1 ACE	20.02	10,934	20.46	9,489	20.22	7,476
2-3 ACEs	29.76	10,934	29.43	9,489	29.68	7,476
4+ ACEs	16.16	10,934	15.97	9,489	15.41	7,476
**Health outcomes**						
Health status in 2013 (5-quantile)			3.007 (1.392)	9,489	3.079 (1.378)	7,476
Health status in 2015 (5-quantile)	2.907 (1.383)	10,934			2.910 (1.361)	7,476
**Adulthood characteristics**						
Educational attainment					6.154 (4.016)	7,476
Personal annual income in 2015					10,276.53 (22,518.34)	7,476

*The unit of personal income is RMB. The “2013–2015 sample” includes respondents who participated in all three waves of the survey*.

### Childhood Circumstances and Health Status in Middle and Later Life

Table 3 examines the relationship between childhood circumstances and health in middle and later life. To verify the stability of the main findings, we regressed four models. The first and second models used the respondents' 2015 health status as the dependent variable, while the third and fourth models used 2013 health status data. We also tried different ways to measure the dependent variable. The first and third models used the ordinary least squares model, which regarded health status as a continuous variable. The second and fourth models adopted the order logit model, which regarded health status as an ordered variable. In each model, we controlled for age and gender, and all results were similar. As shown in Table 3, the five domains of childhood circumstances all have a significant association with health status in middle and later life. In all cases, good health in middle and later life is significantly associated with five variables: having better self-reported health in childhood, having a higher childhood family financial status, the father having a higher education level, and the mother and father having increased longevity. Poor health in middle and later life is significantly associated with three variables: having experienced hunger in childhood, inconvenience in visiting a doctor, and any ACE. All seven of these variables are statistically significant at the 1% level.

**Table 3:** Childhood circumstances and health status in middle and later life.

**Variable**	**Model 1**	**Model 2**	**Model 3**	**Model 4**
	**In 2015 (OLS)**	**In 2015 (OLOGIT)**	**In 2013 (OLS)**	**In 2013 (OLOGIT)**
**Self-reported health status, before age 15 (Poor as the base)**				
Fair	0.605^***^	0.647^***^	0.450^***^	0.505^***^
	(0.057)	(0.057)	(0.055)	(0.061)
Good	0.663^***^	0.795^***^	0.559^***^	0.687^***^
	(0.059)	(0.059)	(0.057)	(0.063)
Ever experienced hunger, from birth to age 5	−0.120^***^	−0.149^***^	−0.100^***^	−0.151^***^
	(0.037)	(0.036)	(0.036)	(0.039)
**Family financial status, before age 17 (Worse as the base)**				
Fair	0.314^***^	0.299^***^	0.310^***^	0.333^***^
	(0.039)	(0.038)	(0.037)	(0.040)
Better	0.428^***^	0.441^***^	0.392^***^	0.476^***^
	(0.065)	(0.064)	(0.064)	(0.070)
Mother's educational attainment	0.014	0.017^*^	0.012	0.015
	(0.009)	(0.009)	(0.009)	(0.010)
Father's educational attainment	0.017^***^	0.019^***^	0.016^***^	0.017^***^
	(0.006)	(0.006)	(0.006)	(0.006)
Received any vaccinations, before age 15	0.186^***^	0.151^***^	0.104^**^	0.070
	(0.053)	(0.052)	(0.050)	(0.054)
Experienced inconvenience in visiting a doctor, from birth to age 15	−0.398^***^	−0.389^***^	−0.400^***^	−0.419^***^
	(0.063)	(0.062)	(0.060)	(0.065)
**Adverse childhood experiences (0 Aces as the base)**				
1 ACE	−0.114^**^	−0.185^***^	−0.091^*^	−0.171^***^
	(0.050)	(0.048)	(0.048)	(0.051)
2-3 ACEs	−0.270^***^	−0.336^***^	−0.258^***^	−0.374^***^
	(0.045)	(0.043)	(0.043)	(0.046)
4+ ACEs	−0.585^***^	−0.671^***^	−0.445^***^	−0.637^***^
	(0.054)	(0.053)	(0.052)	(0.057)
Mother's longevity, in 2015/2013	0.007^***^	0.006^***^	0.004^***^	0.004^***^
	(0.001)	(0.001)	(0.001)	(0.001)
Father's longevity, in 2015/2013	0.006^***^	0.005^***^	0.005^***^	0.005^***^
	(0.001)	(0.001)	(0.001)	(0.002)
Mother still living, in 2015/2013	0.110^**^	0.039	0.080^*^	0.128^***^
	(0.049)	(0.047)	(0.046)	(0.050)
Father still living, in 2015/2013	−0.019	−0.001	0.004	−0.014
	(0.060)	(0.058)	(0.054)	(-0.058)
Age, in 2015/2013	−0.042^***^	−0.045^***^	−0.031^***^	−0.033^***^
	(0.002)	(0.002)	(0.002)	(0.002)
Gender	−0.632^***^	−0.651^***^	−0.494^***^	−0.620^***^
	(0.035)	(0.035)	(0.034)	(0.037)
Constants	1.094^***^	Yes	0.961^***^	Yes
	(0.209)	Yes	(0.202)	Yes
*N*	10,934	10,934	9,489	9,489
*R*^2^/Pseudo *R*^2^	0.1197	0.0453	0.0975	0.0372
**Incremental contribution of variables to** ***R***^**2**^**/Pseudo** ***R***^**2**^				
Childhood health and nutrition	0.0127^***^	0.0059^***^	0.0110^***^	0.0045^***^
Childhood SES	0.0086^***^	0.0033^***^	0.0096^***^	0.0035^***^
Access to health care	0.0044^***^	0.0014^***^	0.0048^***^	0.0015^***^
Parental genetics	0.0055^***^	0.0013^***^	0.0031^***^	0.0014^***^
Adverse childhood experiences	0.0101^***^	0.0049^***^	0.0081^***^	0.0047^***^

*Standard errors in parentheses*.

* *p < 0.1*,

** *p < 0.05*,

*** *p < 0.01*.

The main differences between these four models are as follows. First, the association between the mother's education and health in middle and later life is also significant but only occurs in model 2 (coefficient 0.017, *p* < 0.1). Second, having received any vaccinations is positively and significantly associated with good health in adulthood. However, it is not significant in model 4 (coefficient 0.070, *p* > 0.1), and there are statistically significant differences in the other three models. Third, there is a statistically significant difference within the ACE domain (1 ACE group). Compared with no ACE, one ACE shows a significant association with health in adulthood. In models 2 (coefficient −0.185, *p* < 0.01) and 4 (coefficient −0.171, *p* < 0.01), this association is significant at the 1% level, but, in models 1 (coefficient −0.114, *p* < 0.05) and 3 (coefficient −0.091, *p* < 0.1), it is significant at the 5 and 10% levels, respectively.

To assess how much of the variance in health is explained by different domains of childhood circumstances, we classified these 11 variables into five domains and calculated the incremental value of the *R* square (pseudo *R* square) when each domain was added to the regression with all the other domains. The results obtained are shown at the bottom of Table 3. Among these domains of childhood circumstances (take model 1 as an example), those measuring childhood health and nutrition (Δ*R*^2^ = 0.0127, *p* < 0.01) show the most considerable incremental contribution to the sum of R square. The subsequent order is ACE (Δ*R*^2^ = 0.0101, *p* < 0.01) and childhood socioeconomic status (Δ*R*^2^ = 0.0086, *p* < 0.01), and the last is access to healthcare (Δ*R*^2^ = 0.0044, *p* < 0.01) and parental genetics (Δ*R*^2^ = 0.0055, *p* < 0.01). The Wald and LR tests were used to verify statistical significance when adding each domain to the regression. The results strongly suggest that these explanatory variables should be included in the unconstrained model, and they are all significant at the 1% level. Overall, in our models, childhood circumstances are significantly associated with health status in middle and later life, and the effects of demographic variables are also consistent with expectations.

### The Mechanism of Childhood Circumstances

Table 4 presents results from ordered logits of health status in middle and later life on childhood circumstances. Column 2 presents the results from the reduced form analysis. Except for the second column, each column added new control variables to the model in the front column. Model 5 included educational attainment and personal annual income in 2015, and model 6 further included the respondents' health status in the previous wave. As mentioned above, it is necessary to consider multiple potential mediators when studying the relationship between childhood circumstances and health in later life. For this reason, in our final regression model, we chose to use health status in the previous wave as the proxy variable of the potential missing variables to solve the problem of endogeneity.

**Table 4:** Order logit regression of health status in middle and later life on childhood circumstances and adulthood characteristics.

**Variable**	**Model 4**	**Model 5**	**Model 6**
	**Base case**	**Included education and personal income in 2015**	**Included health status in 2013**
	**2014–2015 sample**	**2014–2015 sample**	**2013–2015 sample**
**Self-reported health status, before age 15 (poor as the base)**			
Fair	0.620^***^	0.606^***^	0.371^***^
	(0.070)	(0.070)	(0.073)
Good	0.787^***^	0.778^***^	0.448^***^
	(0.072)	(0.072)	(0.075)
Ever experienced hunger, from birth to age 5	−0.125^***^	−0.109^**^	−0.036
	(0.044)	(0.044)	(0.046)
**Family financial status before age 17 (worse as the base)**			
Fair	0.342^***^	0.310^***^	0.164^***^
	(0.046)	(0.046)	(0.048)
Better	0.471^***^	0.360^***^	0.173^**^
	(0.079)	(0.080)	(0.082)
Mother's educational attainment	0.011	−0.002	−0.010
	(0.011)	(0.011)	(0.011)
Father's educational attainment	0.022^***^	0.010	0.005
	(0.007)	(0.007)	(0.007)
Received any vaccinations, before age 15	0.111^*^	0.027	0.082
	(0.063)	(0.064)	(0.066)
Experienced inconvenience in visiting a doctor, from birth to age 15	−0.354^***^	−0.324^***^	−0.178^**^
	(0.076)	(0.076)	(0.080)
**Adverse childhood experiences (0 Aces as the base)**			
1 ACE	−0.147^**^	−0.151^***^	−0.075
	(0.058)	(0.058)	(0.060)
2-3 ACEs	−0.323^***^	−0.316^***^	−0.148^***^
	(0.052)	(0.052)	(0.054)
4+ ACEs	−0.702^***^	−0.684^***^	−0.462^***^
	(0.065)	(0.065)	(0.068)
Mother's longevity, in 2015	0.005^***^	0.005^***^	0.002
	(0.002)	(0.002)	(0.002)
Father's longevity, in 2015	0.006^***^	0.005^***^	0.002
	(0.002)	(0.002)	(0.002)
Mother still living, in 2015	0.117^**^	0.095^*^	0.078
	(0.055)	(0.055)	(0.057)
Father still living, in 2015	0.069	0.095	0.140^**^
	(0.067)	(0.067)	(0.069)
Educational attainment		0.040^***^	0.021^***^
		(0.006)	(0.006)
Personal annual income, in 2015 (above the median as the base)		0.402^***^	0.214^***^
		(0.043)	(0.045)
**Health status in 2013 (1st decile of health index as the base)**			
2nd decile of the health index			1.373^***^
			(0.076)
3rd decile of the health index			2.427^***^
			(0.079)
4th decile of the health index			3.257^***^
			(0.083)
5th decile of the health index			4.402^***^
			(0.090)
Age in 2015	−0.038^***^	−0.037^***^	−0.023^***^
	(0.003)	(0.003)	(0.003)
Gender	−0.631^***^	−0.465^***^	−0.256^***^
	(0.042)	(0.045)	(0.047)
Constants	Yes	Yes	Yes
*N*	7,476	7,476	7,476
Pseudo *R^2^*	0.0414	0.0478	0.1839

*Standard errors in parentheses*.

* *p < 0.1*,

** *p < 0.05*,

*** *p < 0.01*.

The results in model 5 show that the father's education level (coefficient 0.010, *p* > 0.1) and the respondent's childhood vaccination status (coefficient 0.027, *p* > 0.1) become insignificant when educational attainment (coefficient 0.040, *p* < 0.1) and personal annual income (coefficient 0.402, *p* < 0.1) are added to the reduced model. Beyond these two variables, the other variables remain significant, but the absolute values of coefficients decline slightly. It appears that socioeconomic status can be identified as a potential mediator between some childhood circumstances and middle-aged and elderly health. For example, the effect of vaccination and the father's level of education is absorbed by the individual's socioeconomic status in later life, and this effect works indirectly on health through an individual's current socioeconomic status.

The results of model 6 show that having experienced hunger in childhood (coefficient −0.036, *p* > 0.1), parents' longevity, and 1 ACE (coefficient −0.075, *p* > 0.1) become insignificant when the individuals' health status in the previous wave is added to the regression. In contrast, childhood self-reported health, childhood family financial status, inconvenience in visiting a doctor (coefficient −0.178, *p* < 0.05), and more than one ACE remain jointly significant predictors of health in later life. Furthermore, the absolute values of coefficients for childhood circumstances are in substantial decline, which indicates the existence of other potential mediators. We should be cautious in explaining the significant relationship between the respondents' current health and their health in the last wave. In our model, we assumed that the recursive nature of the health stock is the most important (10, 48). Therefore, this positive relationship can be explained as the result of other unobserved mediators.

## Discussion

This study focuses on an essential issue: do childhood circumstances, consisting of five domains, have lasting effects on health status in middle and later life? Using three-wave panel data from CHARLS, we found that an extensive set of childhood circumstances were independently associated with health status among the middle-aged and elderly. For example, childhood health and childhood family financial status have a direct impact on health in later life, and they also indirectly impact adult health by affecting socioeconomic status in the same period. Another example is the father's education, as well as experiencing hunger and vaccinations in childhood, indirectly affect adult health through mediators, such as socioeconomic status in the same period. Moreover, this result indicates that some childhood circumstances also contribute to future resources that could enhance or mitigate this effect. Our study also shows that there is relative importance among different domains of childhood circumstances. We should pay extra attention to these three domains: early childhood nutrition and health, family socioeconomic status, and adverse childhood experiences.

Theories and previous studies suggest that a particular domain of childhood circumstances can lead to differences in physical, mental, and cognitive health in adulthood (9, 14, 41, 46). Therefore, the first step of our study focused on whether childhood circumstances are associated with health in middle and later life. First, we applied principal component analysis to 25 health-related questions and constructed a latent index of health status. Considering that most of the input indicators are dichotomous variables, this health index has both continuous and discrete characteristics. Thus, we converted it into a quintile ordinal form. We believe this comprehensive measurement of health status can reflect more information than the commonly used single dimension indicator or “0–1” discrete constructs in the literature (45). In previous research, there is also a measurement that standardizes the averages of multiple health-related indicators to obtain a health score (13). In contrast, our approach can better reflect the differential contribution of indicators to the health index, which means some indicators may have higher weights in explaining health status.

We selected 11 environmental factors in early life as crucial independent variables and formulated them into five domains based on previous experience from the literature. Previous research has emphasized that there may be correlations and cumulative effects between factors in the same domain (20). In our results, we also found this phenomenon. For example, in model 6, only when the events are accumulated to a certain number do adverse childhood experiences have a direct effect on health in later life. Furthermore, this accumulated effect may not appear in the form of a dose-response. Similarly, one domain may be related to others (32), so failure to consider related domains may overestimate the impact of a single domain on health. Therefore, compared to examining each domain in isolation, we tested the interaction between domains and considered them simultaneously in a model. According to the regression coefficients and variance in Table 3, all five domains of childhood circumstances are associated with health in middle and later life, which all remain jointly significant after including them in the same model. The influence of childhood health and nutrition is more prominent and is the most considerable incremental value contributing to the variance in health.

Several hypotheses have been formulated to explain the association between early life experiences and adult health (18, 31, 49). Our research provides new evidence for these pathway models based on data collected from the middle-aged and elderly population in China. Consistent with the life-course model, childhood health, childhood family financial status, inconvenience in visiting a doctor, and more than one adverse childhood experience all have a sustained effect on the health of middle-aged and older adults. Consistent with the results of the Whitehall study, the effect of the father's education or vaccination on health in later life is absorbed by the socioeconomic status over the same period (29). This effect appears to work by impacting the individual's educational attainment and personal income in adulthood. Moreover, socio-economic achievements in later life may partially offset the negative impact of childhood circumstances on health in the same period. In addition, the results in models 5 and 6 also illustrate the universality of potential mediators. Our research shows that the effects of experiencing hunger in childhood, parental longevity, and one adverse childhood experience on health in middle and later life are no longer significant after including the previous health status. It appears that in addition to socioeconomic status, there may be potential mediators that form other health pathways. More importantly, the coefficients in model 6 drop considerably, which shows that mediators greatly reduce the direct effect of childhood circumstances and suggests that these results may be misestimated without accounting for indirect effects via mediators. It overestimates the direct effects of childhood circumstances and misses the indirect pathways of childhood circumstances and their impact on health-related resources. Therefore, investments in children's health not only contribute to health in later life but also dynamically improve the individual's educational attainment and personal income, as well as other life prospects. All these returns may extend far beyond childhood and continue throughout the lifespan.

Some specific policy suggestions are now discussed relevant to those study results mentioned above. First, we should avoid further expanding disparity among children in different groups due to cumulative effects. On the one hand, the state should build a unified child welfare supply system and encourage private resources to supplement public relief projects. On the other hand, the root of the disparity among children lies in their families' poverty (50). Flexible and diverse methods should be adopted to help low-income families improve their socio-economic status. Second, any investment in childhood human capital requires follow-up support measures. For the elderly who have already withdrawn from the labor market, we should pay attention to the social security system's income redistribution effect and increase the level of pensions and pension subsidies for low-income elderly. For the elderly who are not well-educated, we can explore targeted health-related education ways, such as hold community health lectures. Third, we should continue to promote the “integration of medical and elderly care” policy in China, aiming to simultaneously improve healthcare equity and living care quality for the middle-aged and elderly (51).

However, our study has limitations. First, it is necessary to evaluate the accuracy of the data. CHARLS is a retrospective study, and health indicators and critical variables were obtained in the form of self-reports. There may be potential bias when using retrospective questions, either from recall difficulty or deliberate concealment. Therefore, some indicators may be underestimated. For example, when a question involves details from one's distant past, it is often difficult to recall it accurately. Our concern is that underreporting of negative childhood exposures might cause testing errors and impact the significance of variables. Second, for parents' educational attainment, we used a proxy measurement to represent the socioeconomic status of the family. Thus, it may be more appropriate to choose only the father's level of education with time characteristics or change the measurement of the mother's education level to a discrete variable (i.e., distinguish between illiterate and non-illiterate). However, considering the consistency of the data structure, we retain the mother's education level as a continuous variable in our model. Third, in the final model, we matched the data from three waves, so we lost a considerable number of non-equilibrium samples, which only have data recorded in one or two waves. According to the official CHARLS report, during the period between the 2014 life history survey and the 2015 wave, 672 respondents attended no follow-up visits due to death, while 3,265 respondents were lost to follow-up for unknown reasons (38). Therefore, the data may have a sample selection bias. To partially resolve this issue, we separately estimated two sets of samples to check for cohort differences. However, the sample selection problem may have occurred before the 2013 survey. Fourth, questions about the specific period when childhood factors occur and subjective feelings about these experiences are not included in the survey. For example, in addition to the experience of hunger in childhood, the time ranges given by the remaining variables are relatively broad. If we focus the analysis on an earlier stage of childhood, it will help us to understand these phenomena better.

## Conclusions

Our findings emphasize the importance of the life course perspective in studying the impact of childhood circumstances on future health. As this paper has shown, several domains of childhood circumstances have significant effects on health in middle and later life, either directly or indirectly. The results presented in this paper also highlight the role of mediators in the relationship between childhood circumstances and health in later life. The effects of childhood circumstances are passed on to health in later life through their influence on these mediators. An important priority for future research is to identify additional potential mediators. Overall, our findings suggest more attention be paid to investing health capital in early life, and the benefits of these investments will affect the entire lifespan.

## Data Availability Statement

Publicly available datasets were analyzed in this study. This data can be found at: China Health and Retirement Longitudinal Survey. (2020). Available online at: http://charls.pku.edu.cn/pages/data/111/zh-cn.html (accessed April 13, 2020).

## Ethics Statement

An ethics waiver request was submitted to the Ethics Review Board of China Pharmaceutical University, and met the requirements for exemption, as the research relied exclusively on secondary use of anonymous information.

## Author Contributions

TT, YH, and RS: conceptualization, writing—review, and editing. TT: analysis and interpretation of data. TT and YH: writing—original draft preparation. All authors have read, agreed to the published version of the manuscript, and contributed to this manuscript.

## Conflict of Interest

The authors declare that the research was conducted in the absence of any commercial or financial relationships that could be construed as a potential conflict of interest.
